# Technical and Clinical Outcomes of Laparoscopic–Laparotomic Hepatocellular Carcinoma Thermal Ablation with Microwave Technology: Case Series and Review of Literature

**DOI:** 10.3390/cancers16010092

**Published:** 2023-12-24

**Authors:** Riccardo Muglia, Paolo Marra, Domenico Pinelli, Ludovico Dulcetta, Francesco Saverio Carbone, Alessandro Barbaro, Antonio Celestino, Michele Colledan, Sandro Sironi

**Affiliations:** 1 Department of Radiology, Papa Giovanni XXIII Hospital, 24127 Bergamo, Italy; pmarra@asst-pg23.it (P.M.); ldulcetta@asst-pg23.it (L.D.); fcarbone@asst-pg23.it (F.S.C.);; 2 School of Medicine, University of Milano-Bicocca, 20126 Milano, Italya.celestino1@campus.unimib.it (A.C.); 3 Department of General Surgery, Papa Giovanni XXIII Hospital, 24127 Bergamo, Italy; dpinelli@asst-pg23.it (D.P.); mcolledan@asst-pg23.it (M.C.)

**Keywords:** thermal ablation, hepatocellular carcinoma, laparoscopy, laparotomy, microwave ablation

## Abstract

**Simple Summary:**

Although the percutaneous approach has always been the standard to ablate hepatocellular carcinomas (HCCs), both intraoperative laparoscopic and laparotomic approaches have recently demonstrated viability in patients unfit for hepatic resection or percutaneous ablation. Most published papers are based on radiofrequency technology performed with a laparoscopic approach, and the literature lacks up-to-date data on laparotomic microwave ablations. This study discusses the efficacy and safety of intraoperative ablation of HCC with state-of-the-art microwave technology in both laparoscopic and laparotomic fashions, demonstrating high clinical success rates and rare complications for intraoperative HCC ablation unsuitable for a percutaneous treatment, strengthening its performance with microwave technology. Moreover, for the first time in literature, both laparoscopic and laparotomic fashions are reported altogether.

**Abstract:**

Purpose: To evaluate technical and clinical outcomes of intraoperative (laparoscopic/laparotomic) microwave ablation on HCC. Materials and Methods: This is a retrospective single-center study evaluating consecutive patients treated for very early/early-stage HCC with intraoperative microwave ablation from 1 July 2017 to 30 June 2023. In these patients, a percutaneous US-guided approach was excluded due to the nodule’s suboptimal visibility or harmful location and liver resection for a deep position or adherences. Data about the clinical stage, surgical approach, liver pathology and nodules characteristics, technical success, complications, and follow-up were collected. Technical success was intended as the absence of locoregional persistence at follow-up CT/MRI controls. Results: A total of 36 cirrhotic patients (M:F = 30:6, median age 67 years) were enrolled; 18/36 (50%) had a single nodule, 13/36 (36%) had two, 4/36 had three (11%), and 1/36 had four (3%). Among the patients, 24 (67%) were treated with laparoscopy, and 12/36 (33%) with a laparotomic approach. Sixty HCCs of 16.5 mm (6–50 mm) were treated for 7 min (2–30 min) with 100 W of power. A total of 55 nodules (92%) were treated successfully and showed no residual enhancement at the first postoperative follow-up; the other 5/60 (8%) underwent chemo/radioembolization. There was one complication (3%): a biliary fistula treated with percutaneous drainage and glue embolization. The average hospital stay was 3.5 days (1–51 days), and patients were followed up on average for 238 days (13–1792 days). During follow-up, 5/36 patients (14%) underwent liver transplantation, 1/36 (2%) died during hospitalization and 1 after discharge. Conclusions: Laparoscopic/laparotomic intraoperative HCC MW ablation is feasible in patients unsuitable for percutaneous approach or hepatic resection, with rare complications and with good technical and clinical outcomes.

## 1. Introduction

Hepatocellular carcinoma (HCC) represents 75–85% of all liver cancer, the third most common cause of cancer-related mortality, and one of the leading causes of death in cirrhotic patients. The prognosis of HCC is poor, and the mortality rate has been proximal to the incidence rate until the last decade [1].

HCC occurs in up to 90% of cases of a cirrhotic liver, and hepatitis B virus (HBV) is often associated with HCC also in non-cirrhotic livers. However, the epidemiology of HCC is rapidly evolving, with a decrease in viral hepatitis-related cases and an increase in alcohol and nonalcoholic fatty liver disease (NAFLD), fibrosis, and cirrhosis [2]. Moreover, the management of HCC has been improved, especially in the early stages, leading to a decrease in mortality, as even the Barcelona Clinic Liver Cancer (BCLC) staging system has been recently updated to better characterize available treatment options; for these reasons, the accelerated introduction of new therapeutic techniques is expected to lead to favorable prospects [3].

In recent years, surveillance strategies in cirrhotic patients at risk of developing HCC have led to the early diagnosis of the disease. Indeed, patients in the early stages demonstrated higher chances of curative treatments with different options [4]. A number of staging systems are available for HCC, and actually, there is no worldwide consensus on the preferred one. For example, the Child–Pugh system and the model for end-stage liver disease score assess the severity of liver disease, though not including performance status or eventual cancer-related symptoms. Today, the only staging system going beyond these concerns is the BCLC classification, allowing for a more appropriate treatment strategy for each tumor stage [4]: the treatment concept is curative in both the very early (BCLC stage 0) and early stages (BCLC stage A), while palliative for more advanced stages (BCLC stages B, C, and D). Curative approaches account for liver resection, transplantation, or local ablative therapies; palliatives, on the other hand, include transarterial chemoembolization (TACE), radioembolization (TARE), or medical systemic therapies. 

Even large tumors can benefit from surgical resection, and its indication should not be limited by tumor size alone, as long as no vascular invasion is demonstrated and the prediction of the postoperative remnant liver function is adequate [5]. 

Although transplantation represents the main and most important curative option, less than 20% of patients suffering from HCC are eligible for such treatment, fulfilling MILAN criteria or the “extended criteria for liver transplantation”, also after responding to local therapy for tumor downsizing. Moreover, organ donor shortage remains the main limit of liver transplantation [6].

Ablative therapies for HCC are classified as chemical ablation (i.e., ethanol) or thermal ablation (i.e., radiofrequency (RF), microwave (MW), cryoablation, and high-intensity focused ultrasound) [7]. In particular, ablation techniques are less invasive treatments compared to surgery due to the possibility of performing a percutaneous approach, using imaging guidance such as ultrasonography (US) or computed tomography (CT); another advantage offered by ablation is the possibility of easily repeating the procedure, in order to achieve better outcomes or to treat local disease persistence [7]. Furthermore, studies suggest that RF ablation might be capable of stimulating the anti-tumor immune response in HCC patients, even if the response is insufficient to prevent tumor recurrence [8].

Thermal ablation has gained wide acceptance in the management of HCC, but high rates of clinical success have also been demonstrated for various other liver solid tumors, such as intrahepatic cholangiocarcinoma and liver metastases from neuroendocrine tumors, colorectal cancer, and breast cancer [9,10], as well as in different anatomical sites, such as kidney (renal cell carcinoma and reninoma [11,12]) or lung (primary or metastatic lesions [13,14]); in the kidney, in particular, ablation showed to be effective also when compared to surgery [15], significantly expanding its impact in the field of interventional oncology. 

In patients with early HCC, RF has demonstrated similar overall survival to hepatic resection, along with lower morbidity rates due to its minimally invasive nature [16]. However, the surgical option is often considered as the treatment of choice in the context of preserved liver function. This is mainly attributed to relatively high rates of local recurrence, varying from 10% to 40% within 5 years from ablation, mainly depending on tumor size and nodules’ number [17]. Yet, despite recent developments and technical improvements in device design (such as internal cooling and multi-tined electrodes) [18] and energy delivery systems (like electrode-switching activation and pulsed energy delivery), RF has been historically associated with a number of restraints, including long ablation times, heat-sink effect adjacent to blood vessels, and tissue charring around the tip of the electrode. In addition, suboptimal energy delivery of old RF generators limits the effective ablation volume when exposing an electrode tip of more than 2.5–3.0 cm due to the lack of coagulative necrosis sphericity [19].

Recently, a more thorough and systematic optimization of the RF pulsing algorithms has been reported [20], allowing for the administration of a greater amount of energy to liver parenchyma for tip exposures of up to 5 cm and associated with spheric necrotic volumes. However, despite the technological improvements, RF is being replaced by MW technology worldwide, allowing for higher intra-tumoral temperatures, larger ablative volumes, and faster ablation times. In addition, MW is less sensitive to the heat sink effect compared to RF [20].

For ablations performed under US guidance, the probe is introduced percutaneously inside the liver through an epigastric or intercostal access, and the tip is placed at the deepest margin of the target tumor. Then, the ablation is performed with time and wattage according to HCC dimensions: multiple overlapping ablations are sometimes required to achieve complete necrosis for nodules > 3 cm. However, it can be challenging to ablate HCCs under US guidance when nodules have poor sonographic conspicuity [7]. 

Although the percutaneous approach has been regarded for years as the standard to ablate liver lesions, both intraoperative laparoscopic and laparotomic approaches have recently been demonstrated viable in a significant proportion of patients judged unfit for hepatic resection or percutaneous ablation [21]. Indeed, some HCC nodules < 3 cm in diameter should be treated with minimal invasiveness, but for their location in the hepatic dome or in subcapsular locations, the standard imaging-guided percutaneous ablation might be harmful. Some papers have demonstrated that HCCs located near the capsule relapse more frequently than deeper nodules because of little ablation safety margins. On the other hand, during percutaneous ablation of deeper nodules, adjacent visceral organs can be damaged or perforated due to inaccurate US visualization. Moreover, seeding of malignant tissue to the outside of the tumor can happen while ablating subcapsular lesions, even if the tumor is not punctured directly. So, the intraoperative approach has been developed not only to ablate nodules that are relatively inaccessible percutaneously but also to prevent damage to adjacent organs during the procedure [22]. Thus, laparoscopy or laparotomy can be advantageous approaches to overcome some limitations of US or to ablate tumors that are inaccessible percutaneously, although losing minimal invasiveness [23].

Across the last decade, some papers have been published on intraoperative thermal ablation. However, most of them report only on thermal ablations performed with RF technology, lacking up-to-date data on MW advancement. Moreover, published data are based on laparoscopy and do not take into account the possibility of a switch to a laparotomic approach. For this reason, this study aims to review our cohort of patients undergoing intraoperative HCC ablation, with the aim to evaluate outcomes on both laparoscopic and laparotomic approaches performed only with state-of-the-art MW technology and to discuss them in comparison with those previously published on both RF and MW. Moreover, as many imaging-guidance alternatives are actually available to overcome US limits, this study investigates the main differences between them and intraoperative ablation.

## 2. Material and Methods

### 2.1. Study Population

This study was conducted according to the guidelines of the Declaration of Helsinki and approved by the local ethics committee (protocol no. 132-21). Informed consent for anonymous data review and publication was obtained from all individual participants included.

Consecutive patients treated from 1 July 2017 to 30 June 2023 by intraoperative thermal ablation for HCC in a single tertiary referral center for liver diseases (ASST Papa Giovanni XXIII, Bergamo, Italy) were retrospectively reviewed.

All of the HCCs were diagnosed through a non-invasive radiological work-up, following the European Association of the Study of the Liver (EASL) 2018 clinical practice guidelines [24].

All patients received an indication for intraoperative ablation based on a multidisciplinary discussion among liver surgeons, gastroenterologists, and interventional radiologists. HCC visibility was assessed with US and contrast-enhanced US (CEUS) prior to ablation and, in all cases, tumor conspicuity was poor to absent, whereas contrast-enhanced CT or contrast-enhanced MRI clearly depicted all nodules with hallmarks of HCC. 

For these reasons, the inclusion criteria for this study were as follows: (1) participants aged 18 years or older; (2) patients suffering from very early/early-stage HCC, unsuitable for liver transplant; (3) patients with small liver remnants or other contraindications to resection; (4) patients evaluated for percutaneous ablation by US/CEUS, proving nodule suboptimal visibility or harmful location; (5) patients treated in a intraoperative fashion, both laparoscopic or laparotomic; and (6) HCCs ablated with MW technology. All the ablations of metastatic liver lesions or those ablations performed with other-than-MW technology were excluded.

Data were collected about liver pathology (i.e., HBV-/HCV-related, metabolic, cryptogenic, alcoholic), surgical approach (i.e., laparotomic or laparoscopic), location (i.e., liver segment), number and dimensions of nodules treated, ablation time and wattage, technical success, complications, discharge date, days of hospitalization, and follow-up.

Complications were reported following the CIRSE classification system [25]. Technical success was intended as the absence of residual diseases at follow-up CT/MRI controls [26].

All procedures were performed by 4 interventional radiologists (with at least 2 years of experience in liver interventional oncology), with MW technology, representing the up-to-date technology, fast and not burdened by the heat-sink effect, if compared to the available RF technology. Cryoablation was not included due to its unavailability at the study center. 

### 2.2. Laparoscopic and Laparotomic Ablation

All intraoperative laparoscopic and laparotomic ablation procedures were performed under general anesthesia.

**Figure 1:**
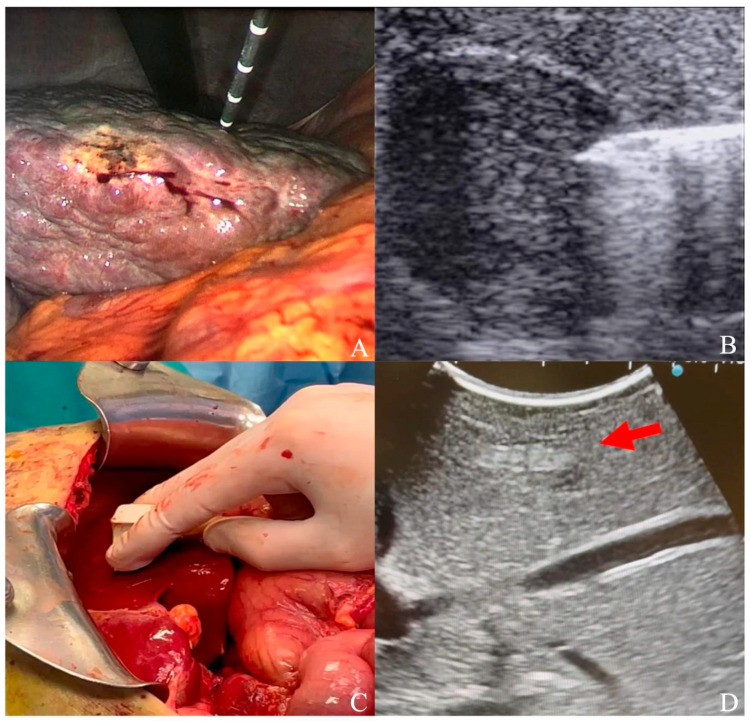
(**A**) Laparoscopic ultrasound-guided thermal ablation with microwave antenna; (**B**) microwave antenna approaching a hypoechoic HCC under laparoscopic ultrasound guidance; (**C**) laparotomic ultrasound; (**D**) hyperechoic HCC (red arrow) visualized under liver surface.

For the laparoscopic approach, abdominal access was performed with a 12 mm optical trocar in the right upper quadrant. The pneumoperitoneum was induced through CO_2_ inflation to maintain 12/14 mmHg of pressure. Subsequently, another 12 mm trocar was placed in the abdominal wall, considering the position of HCC inside the liver (e.g., left lobe nodules required a median access, while a right upper quadrant trocar was preferred for a right lobe nodule) [27]. For laparoscopic liver US, a high-frequency linear probe was advanced through the trocar to the liver surface (Figure 1). Depending on the tumor position and its adjacent tissues, an additional trocar could be added under the xiphoid process or next to the costal margin, aiming to separate adhesions and completely expose the affected liver segment [28]. As the nodule was identified, a percutaneous 30 cm MW probe (Emprint, Medtronic, Minneapolis, MN, USA) was advanced through the abdominal wall to the liver surface under laparoscopic guidance; then, under US guidance, the probe was advanced into the nodule.

To perform a laparotomic ablation, a right or bilateral subcostal incision was generally utilized to expose the liver. With a micro-convex probe (Esaote, Genova, Italy) on the liver surface, segments were explored on the basis of previous CT/MRI until the HCC nodule was identified (Figure 1). Then, a transliver 15/20 cm MW probe (Emprint, Medtronic, Minneapolis, MN, USA) was advanced inside the nodule under US guidance. In both laparoscopic and laparotomic procedures, the ablation was performed at 100 W, with a variable duration on the basis of nodule dimensions according to ablation charts reported in the instructions for use. 

**Figure 2:**
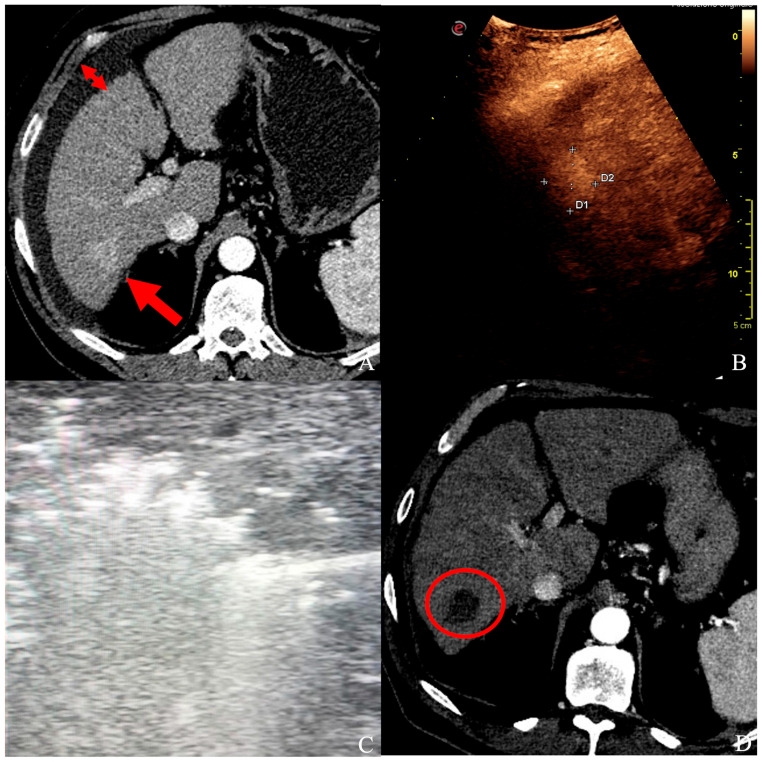
(**A**) A patient with ascites (short arrows) and a 25 × 20 mm HCC in segment VI (long arrow), confirmed by contrast-enhanced ultrasound (**B**), and unsuitable for ultrasound-guided percutaneous ablation. (**C**) Under laparoscopy, HCC was ablated with a single probe deployment, and the whole nodule turned hyperechoic. (**D**) At follow-up CT control, a well-defined necrotic volume with no signs of HCC persistence in segment VI (red circle).

With both approaches, the coverage of the tumor by hyperechoic gas under real-time ultrasound was regarded as a measure of complete tumor ablation (Figure 2); at least 5 mm of the hepatic parenchyma surrounding the tumor was always ablated to guarantee safety margins [26,29]. To treat tumors within 3 cm in diameter, a single ablation with one probe was usually sufficient; for tumors of 3 to 5 cm, multiple overlapping ablations were performed. At the end of every treatment, in spite of the intraoperative approach, track ablation was performed at 100 W until the antenna tip reached the liver surface.

## 3. Results

The BCLC staging system was used to classify all the patients analyzed in our study. According to this, 33 patients suffered from very early or early-stage HCC and initially adhered to the inclusion criteria of this study; 2 patients had nodules measuring ≤ 30 mm on preoperative imaging, but at the intervention were grown up to 40 and 50 mm, respectively; and 1 patient had 3 HCCs on preoperative imaging but a new, forth nodule was detected under laparotomic US. So, 36 patients (M:F = 30:6, median age 67 years, range 52–83 years) with 60 HCCs were finally included in the analysis.

All patients had a clinic-laboratory and instrumental diagnosis of liver cirrhosis, including liver biopsy and liver function tests; in relation to the cause of cirrhosis, this was related respectively to alcohol (16/36, 44%), HCV (8/36, 22%), HBV (5/36, 14%), metabolic (4/36, 11%), multiviral (HBV + HCV, 2/36, 6%), and cryptogenic (1/36, 3%) causes. Among 36 patients, 18 (50%) had a single nodule, 13 (36%) had two, 4 had three (11%), and 1 had four (3%).

As for the technique used to ablate the HCCs, 24 patients (67%) were treated in laparoscopy, and 12 (33%) with a laparotomic approach. The 60 HCCs measured 16.5 mm (median value, range 6–50 mm) and were ablated for 7 min (range 2–30) with 100 W of power. All nodules were treated in the same session. Six nodules (10%) of ≥20 mm needed more than one antenna deployment to overlap ablation volumes and cover the whole tumor. HCCs were located in segments II (3/60, 5%), III (6/60, 10%), IV (12/60, 20%), V (9/60, 15%), VI (4/60, 7%), VII (6/60, 10%), and VIII (20/60, 33%).

**Figure 3:**
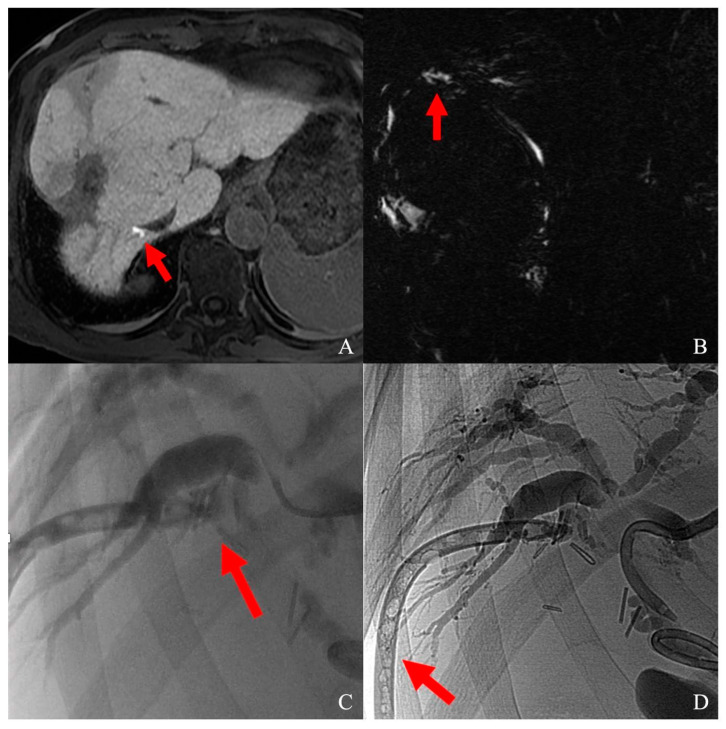
(**A**) Biliary duct lesion after segment VIII HCC laparotomic thermal ablation, visualized as a contrast leak (red arrow) at T1 fat-sat sequence on hepatobiliary phase; (**B**) leak visualization on MRCP (red arrow); (**C**) superselective catheterization of the damaged biliary duct; contrast media flushing from bile duct to abdominal drainage (red arrow); (**D**) sealing of the damaged bile duct; the abdominal drainage was glued as well (red arrow).

Fifty-five nodules (92%) were treated successfully, with no signs of residual diseases in any of the CT/MRs performed during the follow-up; the other five nodules (8%) showed tumor persistence at the first post-operative follow-up; therefore, patients underwent subsequent chemo/radioembolization. Among all cases, only one complication was encountered (3%): a biliary fistula treated with percutaneous biliary drainage and glue embolization (Figure 3).

The average hospital stay was 3.5 days (range, 1–51), and the mean follow-up time was 238 days (range, 13–1792 days). During follow-up, one patient (2%) died during hospitalization, one after discharge, three patients (6%) progressed to the intermediate/advanced HCC stage, and five patients (14%) underwent liver transplantation.

## 4. Discussion

This retrospective study showed that MW can be considered a safe and effective technology to quickly ablate HCC nodules in an intraoperative fashion. Additionally, with this research, up-to-date and relevant data are added to previously published studies about MW ablation, as it first investigates the application of state-of-the-art technology with both laparoscopic and laparotomic approaches.

Currently, ablative therapies have become the third pillar among treatments for HCC nodules and have emerged as an alternative to hepatic resection and liver transplantation for early and very early-stage HCC. However, US-guided percutaneous ablation is reported unfeasible in about 25–55% of patients due to unfavorable HCC location [28]. For example, the complications and local recurrence rates are higher in those nodules located at the liver surface if compared to deep tumors [30]; moreover, subphrenic tumors are hard to clearly depict under percutaneous US and, for this reason, are often judged unsuitable for percutaneous ablation. Wang et al. described a series of 51 patients treated with laparoscopic MW ablation, all with HCC located at the liver surface; they reached a complete ablation rate of 92.2%, with a low complication rate [28]. These results are in line with the ones observed in this study and strongly suggest performing intraoperative MW for those HCCs located in a hard-to-treat position. In these cases, intraoperative ablation can serve as a valid alternative to US guidance, guaranteeing a safer approach to liver tumors whose locations render the percutaneous approach unfeasible or challenging [31]. 

In recent studies comparing different ablation technologies, RF has been demonstrated to be as effective as MW for HCCs up to 3 cm, with 97% in terms of efficacy and 68% as 5-year survival [32], while MW showed a faster ablation procedure, a reduced heat-sink effect and improved convection profile [33]. Overall, the published studies support the comparability of the two technologies in terms of survival, local tumor control, and complication rates, with some notable exceptions, such as nodules proximal to vessels or ≥2.5 cm in diameter, for which MW performs better than RF [34,35]. 

Despite not taking into account the modality of approach, the last meta-analyses and randomized controlled trials describe a 96–98% technical success for MW ablations [36,37], substantially in line with the 92% observed in this study. In particular, the Emprint Ablation System with Thermosphere Technology (Emprint, Medtronic, Minneapolis, MN, USA) was an improved version of Covidien’s previous generation, attempting to overcome the limitations of unpredictable size and shape of the ablation zone. This device performs at 2450 MHz MW and consists of a 100 W generator with a saline pump that provides cooling of the antenna during ablation. The probes consist of a single-body fiberglass shaft that minimizes the risk of fractures and come in various lengths (15, 20, and 30 cm) [38]. The unique properties that allow this product to induce reliable large spherical zones of necrosis rely on thermal control, field control, and wavelength control and have demonstrated shorter ablation times and lower 3-year local tumor progression when compared to RF, especially for subcapsular HCCs [39].

The laparoscopic approach was also described for ablating multiple HCCs [40]. Differently from this cohort, Yoon et al. performed their treatments with RF technology, bearing the necessity to place the electrode in two to six different sites inside tumors >20 mm to overlap necrotic volumes and to obtain maximal tumor coverage. Owing to the need to insert electrodes inside the liver more than once, their population suffered a higher risk of bleeding and biliary complications (13%); moreover, they observed a higher recurrence rate during follow-up when compared to the data from this study (41% vs. 8%); this was possibly due to the weaknesses intrinsic in the use of RF. So, new data strengthen the possibility of safely ablating multiple HCCs simultaneously, although wider nodules could request more than one antenna deployment for both MW and RF. 

An interesting research has recently investigated 815 HCC ablations with the sole laparoscopic MW [41], confirming the safety and minimal invasiveness of this procedure on a high-volume cohort. Despite reporting high survival rates and rare complications, the authors did not take into account those treatments performed with a laparotomic approach, although conversion can generally happen in up to 4% of patients [40]. Focusing on this study’s cohort, up to one-third of laparoscopies were switched to laparotomies, mainly due to adhesive adherence in patients who had undergone previous abdominal surgeries. Indeed, this is one of the principal reasons it was decided to present the whole cohort, considering laparoscopic and laparotomic treatments altogether.

Sorafenib, a multi-targeted kinase inhibitor, is the first medical treatment succeeding in advanced HCC control and represents a breakthrough in the management of this tumor [42]. Some authors recently suggested adding such medical treatment to HCC ablation; although burdened by a high incidence of adverse reactions related to sorafenib, the combined treatment showed longer survival and longer ablation intervals compared to those of MW alone and seems worthy of future research in order to improve ablation success rates [43].

In the presented cohort, the only complication encountered was a biliary fistula dripping bile outside the right liver after the ablation of a 28 mm HCC located in segment VIII. This is a rare event after intraoperative ablation [41] that was detected for the biliary component of drainage fluid some days after treatment. An MRI performed with a hepatobiliary contrast agent (Primovist, Bayer, Germany) confirmed the leak location. To halt the bile duct leak, percutaneous US-guided access to the left bile ducts was performed and crossed contralaterally with a microcatheter (Carnelian, Tokai, Japan). As a contrast leak was detected from the culprit bile duct, the microcatheter was navigated superselectively to the damaged area, and contrast media was injected, flushing from the liver margin inside the abdominal drainage; so, the leak was successfully sealed with glue (Glubran 2, GEM, Viareggio, Italy) mixed to ethiodized oil (Lipiodol, Guerbet, Villepinte, France). 

Conventional CT-fluoroscopy guidance for percutaneous ablation enables a three-dimensional view of the target tumor and its surrounding structures, the antenna pathway, and the post-ablation tissue changes. However, this guiding technique is impaired by important disadvantages, such as high exposure to radiations, limited angulation during the insertion of the antenna, short contrast-enhanced imaging time frame, and suboptimal visualization of intrahepatic vessels and bile ducts; indeed, high volumes of intravenous contrast media might be administered during difficult-to-target ablations, and patients can experienced post-ablation induced nephrotoxicity [44]. In patients with unresectable liver malignancies, invisible on US or unenhanced CT, a single-session CT hepatic arteriography-guided percutaneous tumor ablation enables accurate contrast-enhanced imaging and real-time contrast-enhanced CT fluoroscopy, leading to better lesion conspicuity, with an excellent technical success rate and reduced contrast media volumes [45]. Although avoiding the intraoperative approach for nodules located in challenging positions, the downside of this technique is the need for arterial access, bearing additional complications (i.e., hematoma, pseudoaneurysm, arteriovenous fistula, arterial thrombosis), and logistic issues such as a hybrid operating room with CT/angiography system.

Another imaging modality often combined with percutaneous ablation is cone-beam computed tomography (CBCT). Several studies have shown the effectiveness of using CBCT for immediate treatment assessments and follow-up, and satisfactory outcomes can be achieved [46]. Moreover, it has also been demonstrated how the outcomes of CBCT guidance are similar to those of traditional CT guidance for HCC ablation.

Recently, Cha et al. evaluated the efficacy of fluoroscopy-guided TACE plus RF for the treatment of small HCCs unsuitable for a US-guided RF ablation [47]. Indeed, TACE can be intended as a minimally invasive treatment for tumor control and to improve patients’ survival [48], but by itself cannot be considered as curative, but rather a palliative treatment. Instead, fluoroscopy-guided RF performed after TACE can be a potentially curative treatment in US-invisible nodules: this is because electrode insertion can be accurately performed under fluoroscopic guidance by targeting the nodule to become radiopaque after TACE microparticles deployment [49]. In addition, the decreased blood flow to the HCC nodule may reduce the heat sink effect, increasing the ablation volume [50]. In terms of outcomes, the authors found that the combination of TACE plus RF for HCC was similar to those of standard US-guided RF. However, even if limited by the intrinsic complications of transarterial catheterization and by the doubtful utility of chemotherapeutic agent administration in guiding small HCCs ablation, the secondary increase in necrosis volume can be seen as a valid reason to propose TACE plus RF as an alternative to the intraoperative ablation approach.

Although augmented reality (AR) has been recently proposed to overcome the limits of percutaneous US guidance [51], this kind of new technology seems not ready to substitute the traditional guiding systems [52]; indeed, although rapid system setup and procedural targeting times are reported, the most critical issue for the use of AR in medical applications is the superimposition precision, intended as registration accuracy. Moreover, the patient’s respiratory movement and motion remain one of the largest technical and practical hurdles, as AR guidance systems are currently unable to follow respiratory excursions in mobile organs with real-time corrections, bearing a risk of the shifting of the intended target relative to the expected location [53].

Fusion imaging allows the match of real-time ultrasound images with pre-ablation CT or MRI and is commonly used in a percutaneous fashion. Recently, Luo et al. demonstrated how ultrasound fusion imaging can be also used for thermal ablation in an intraoperative fashion with satisfactory tumor control [54]. In order to improve laparoscopic thermal ablations, Santambrogio et al. reviewed other technologies that have been recently developed to facilitate laparoscopic ablation and to improve the results in terms of radicality and complications [55]. For instance, they identified the use of 3D reconstruction and indocyanine green fluorescence administration as improvement tools that can help show the exact spatial anatomy, plan the ablation, or target the nodule during treatment.

With the advent of new guiding techniques, ablations have nowadays progressed to a surgical level; procedures are less invasive, increasing precision while reducing operating time. However, these technological developments require sophisticated technical support in order to perform the best possible therapy for the best-selected patient. So, interventional radiologists, along with surgeons, are responsible for the evaluation of all the strengths and weaknesses of different approaches [56]. In the near future, interventional radiologists will be asked to master every guidance in order to maximize the clinical effectiveness of ablations, individually tailored for every patient [52].

It is important to consider the main limitations of this research. First, this was a non-randomized observational study, which introduces potential biases in patients’ selection and cohort inhomogeneities. Second, the numerous, less invasive alternative guidance techniques make intraoperative ablation a rare expedient; this led to describing a small population, preventing it from going beyond a descriptive statistical analysis. This study also lacks a long-term follow-up; however, performing in a high-workload tertiary liver transplant center, some of the patients described underwent thermal ablation for downstaging and were transplanted briefly after. Future research should collect prospective data, expand the patient sample, prolong the follow-up, and consider other evaluation parameters, such as disease-free survival or overall survival.

## 5. Conclusions

This study is the first to highlight the value of HCC ablation with the sole MW technology on intraoperative ablations. Both laparoscopic and laparotomic approaches are feasible in patients unsuitable for percutaneous approach or hepatic resection, are burdened by rare complications, and show good technical and clinical outcomes. However, further investigations are necessary to validate these approaches on a large scale. Future interventional radiologists should aim to become experts in every ablation guidance technique in order to overcome US limitations.

## Data Availability

The data presented in this study are available on request from the corresponding author. The data are not publicly available due to privacy reasons.
